# Tibetan Butter Tea Consumption and Cognitive Performance Among Tibetan Adolescents at High Altitude

**DOI:** 10.3390/nu18183017

**Published:** 2026-09-15

**Authors:** Liping Deng, Yuhang Fang, Pingcuo Pingcuo, Cirenyangzong Cirenyangzong, Wenhu Zhao, Junmei Zhao, Yanhui Wang, Simin Xu, Qiguo Lian

**Affiliations:** 1 Lhasa Center for Disease Control and Prevention, Lhasa 850005, China; dengliping@lscdc.xz.cn (L.D.); pingcuo@lscdc.xz.cn (P.P.); cirenyangzong@lscdc.xz.cn (C.C.); zhaowenhu@lscdc.xz.cn (W.Z.); zhaojunmei@lscdc.xz.cn (J.Z.); wangyanhui@lscdc.xz.cn (Y.W.); 2 Shanghai-MOST Key Laboratory of Health and Disease Genomics, Shanghai Institute for Biomedical and Pharmaceutical Technologies, Shanghai 200237, China; yhfang17@fudan.edu.cn

**Keywords:** Tibetan butter tea, cognitive performance, students, high altitude, Xizang

## Abstract

**Background:** Tibetan butter tea sits at a tension between the health concerns raised by its high salt and fat content and the potential cognitive benefits of its tea-derived constituents, yet whether this traditional beverage is associated with cognition in high-altitude Tibetan adolescents has not been directly examined. **Methods:** We conducted a school-based cross-sectional study of Tibetan students in grades 7–12 in Lhasa, Xizang, China, between June 2024 and June 2025. The analytic sample included 7967 students, with ages ranging from 10.18 to 24.82 years (mean ± SD: 16.06 ± 1.80). Butter tea consumption frequency was self-reported. Cognitive performance was assessed with Raven’s Standard Progressive Matrices; low cognitive performance was defined as a score below the grade-specific 25th percentile. Logistic regression with cluster-robust standard errors estimated odds ratios (ORs) and 95% confidence intervals (CIs), adjusting for demographic, family, socioeconomic, sleep, physical activity, and lifestyle factors, both overall and in sex-specific models. **Results:** Among 7967 Tibetan students, 3534 were male, and 4433 were female. Low cognitive performance was most prevalent among students reporting daily (29.56%) or never (26.34%) consumption and less prevalent among those reporting occasional (22.18%) or frequent (20.39%) consumption. Compared with never consumption, occasional consumption (aOR = 0.79; 95% CI, 0.70–0.88; *p* < 0.001) and frequent consumption (aOR = 0.71; 95% CI, 0.55–0.90; *p* = 0.004) were associated with lower odds of low cognitive performance. Daily consumption was not statistically significant. In sex-stratified analyses, frequent consumption was associated with lower odds among males (aOR = 0.68; 95% CI, 0.49–0.93; *p* = 0.018). Among females, both occasional (aOR = 0.76; 95% CI, 0.64–0.90; *p* = 0.001) and frequent (aOR = 0.74; 95% CI, 0.58–0.93; *p* = 0.011) consumption were associated with lower odds. **Conclusions:** Occasional and frequent consumption of Tibetan butter tea was associated with reduced odds of low cognitive performance among Tibetan adolescents at high altitude, with modest sex differences. These findings suggest that Tibetan butter tea should be evaluated as a culturally embedded whole-dietary exposure and may inform culturally sensitive school-based nutrition education and public health guidance in high-altitude Tibetan settings; however, causal inference cannot be established from this cross-sectional study, and longitudinal validation is needed.

## 1. Introduction

Adolescence is a critical sensitive period for neurodevelopment, learning, and academic attainment, during which the normal maturation of cognitive control, executive function, and learning ability is essential for long-term development [1]. Cognitive impairment is a major neurodevelopmental challenge among adolescents worldwide, and intellectual disability is one of its most severe clinical manifestations. The Global Burden of Disease Study 2017 estimated that the prevalence of intellectual disability among adolescents aged 10–14 years and 15–19 years was approximately 3169 and 3083 per 100,000 population, respectively, corresponding to approximately 39.17 million prevalent cases in total (20.16 million among those aged 10–14 years and 19.01 million among those aged 15–19 years) [2]. A meta-analysis of 52 population-based studies further reported a pooled prevalence of 10.37 per 1000 population and showed that the prevalence was higher in children/adolescents than in adults [3]. The vulnerability of neurocognitive development during adolescence has important public health implications.

In high-altitude settings, chronic hypobaric hypoxia may affect the developing brain, and studies among Tibetan and Andean adolescents have reported lower cognitive performance and neurophysiological differences associated with higher-altitude exposure [4,5,6]. Altitude itself, however, is not readily modifiable. Diet represents a potentially actionable factor for supporting cognitive development in this population.

Tibetan butter tea holds a central position in the traditional Tibetan diet. It is made by boiling brick tea and then churning it with yak butter and salt and is consumed with meals almost daily [7,8]. The drink is inseparable from tsampa—the staple food of the Plateau, made by kneading roasted highland barley flour with Tibetan butter tea into a dough before eating [7]. As a staple beverage, butter tea also constitutes an important part of daily fluid intake. This role reflects its original function in cold, high-altitude environments, where long winters, low temperatures, and hypoxia shape distinctive nutritional needs [8]. Amid a rapid nutrition transition, this practice remains widespread: although diversified market foods have entered the Tibetan diet, Tibetan butter tea remains a near-daily staple in many households, as this shift is additive rather than substitutive [7,9,10]. It persists because, as a culturally distinctive primary beverage type [7], it constitutes, together with tsampa, the core of the local traditional dietary pattern and carries indigenous tradition and intergenerational dietary identity [7,10]; continued daily intake can promote taste preferences acquired early in life [11]; and its high energy density matches cold, hypoxic conditions, retaining functional value for populations engaged in farm and herding labor [8,10]. Tibetan butter tea therefore remains an important daily dietary exposure among Tibetan adolescents.

Considered at the component level, Tibetan butter tea presents opposing possibilities for cognition. On the one hand, its tea-derived constituents, particularly L-theanine and caffeine, have been linked in meta-analyses of randomized trials to short-term improvements in attention-related outcomes, suggesting that tea-based beverages may have cognitive benefits [12]. On the other hand, its high salt and fat content raises concerns about potential harm: high dietary sodium has been linked to impaired vascular and cognitive function through endothelial and cerebral perfusion pathways, and high-fat dietary patterns have been associated with inflammation and adverse cognitive outcomes in adolescent populations [13,14,15,16]. Although these component-level lines of evidence provide biological plausibility for an association, they cannot substitute for direct evidence on Tibetan butter tea as a whole food within the target population.

Existing evidence on Tibetan butter tea and health is also outcome-specific. Among Tibetan adults, higher butter tea intake was inversely associated with metabolic syndrome [17], whereas brick tea consumption has been linked to endemic fluorosis in Tibetan areas [18,19]. These contrasting findings illustrate that the health implications of a traditional tea-based beverage cannot be inferred from its nutrient composition alone and must be evaluated separately for each health outcome.

To our knowledge, no published population-based or animal study has directly examined the association between Tibetan butter tea consumption and cognitive performance. Hence, we conducted a school-based cross-sectional study among middle and high school students in Lhasa, Xizang, China. We aimed to examine whether the frequency of Tibetan butter tea consumption was associated with low cognitive performance among high-altitude adolescents.

## 2. Methods

### 2.1. Study Design and Participants

We conducted a school-based cross-sectional study in Lhasa, Xizang, China, between June 2024 and June 2025. Seven general secondary schools were selected using cluster sampling, and Tibetan students in grades 7–12 were surveyed after written informed consent was obtained from both the students and their parents or legal guardians. Their ages ranged from 10.18 to 24.82 years (mean ± SD, 16.06 ± 1.80). Data collection was conducted in classrooms using paper-and-pencil questionnaires during regular school hours and took about 45 min, with the help of trained investigators. Questionnaire data were entered using EpiData Entry 3.1, with double data entry to reduce data entry errors.

**Figure 1:**
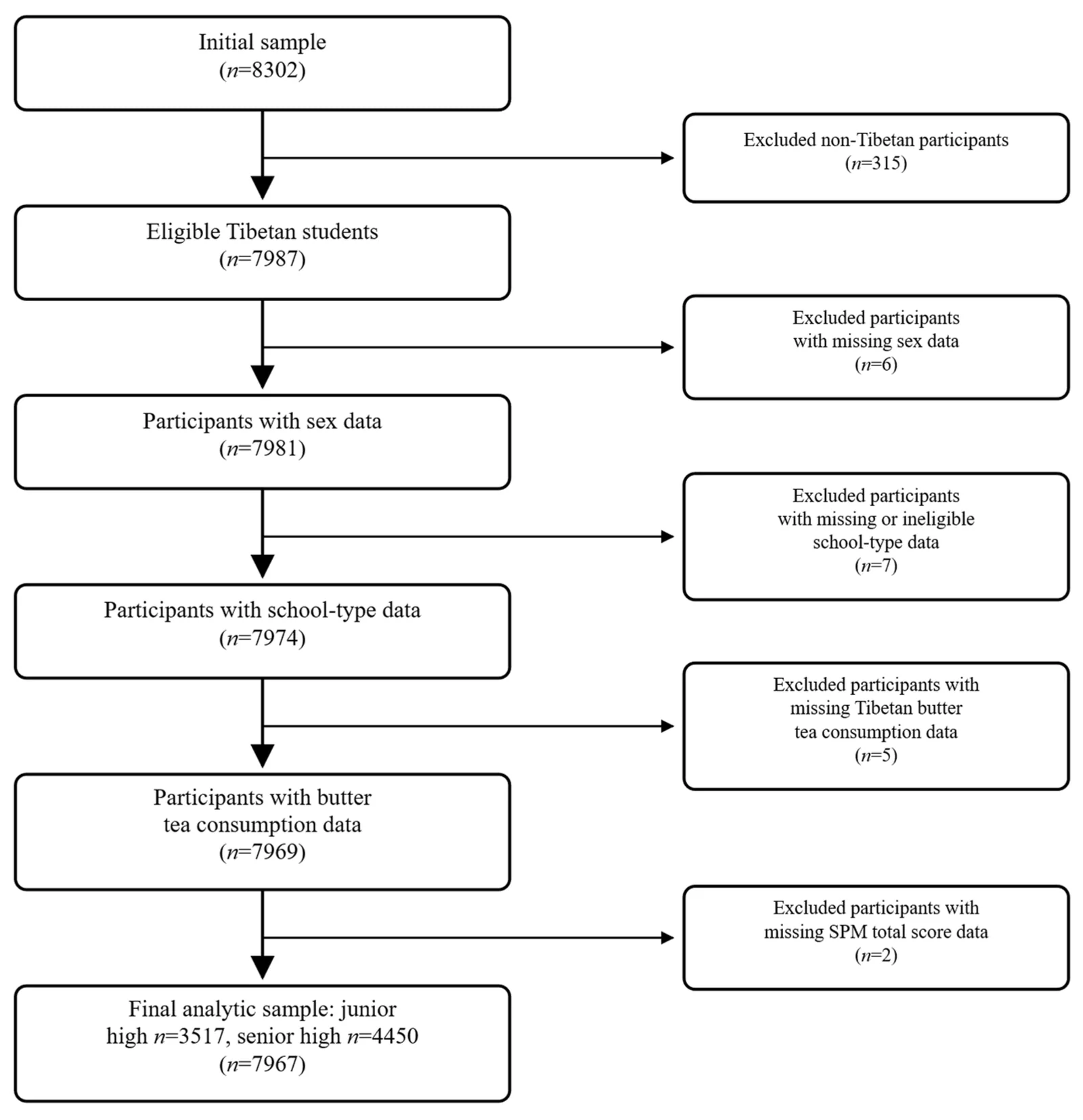
Participant flowchart.

In total, 8302 students completed the survey. Participants were excluded if they were non-Tibetan (*n* = 315), had missing data on sex (*n* = 6), school type (*n* = 7), or Tibetan butter tea consumption frequency (*n* = 5), or had a missing Raven’s Standard Progressive Matrices (RSPM) score (*n* = 2), as shown in Figure 1. The final analytic sample comprised 7967 students, including 3517 junior school students and 4450 senior school students.

### 2.2. Measures

#### 2.2.1. Assessment of Cognitive Performance

Cognitive performance was assessed using RSPM [20]. The RSPM is a 60-item nonverbal test designed to evaluate abstract reasoning and fluid intelligence using geometric figures with a missing piece. The test consists of five sets (A, B, C, D, and E), each with 12 items. The test assesses reasoning processes, including pattern completion, analogical reasoning, detection of progressive changes, spatial transformation and permutation, and abstract relational reasoning. Each correct answer was assigned one point, giving a total score ranging from 0 to 60, with higher scores indicating better performance.

Because the raw RSPM scores are expected to differ across grades, cognitive performance was classified using grade-specific score distributions to reduce misclassification due to developmental and educational differences [20]. Students with RSPM scores below the 25th percentile were considered to have low cognitive performance, while those at or above this threshold were classified as higher cognitive performers [21].

#### 2.2.2. Assessment of Tibetan Butter Tea Consumption Frequency

Tibetan butter tea consumption frequency was assessed using a self-reported dietary frequency item adapted from the Health Behaviour in School-aged Children study questionnaire [22]. Students were asked, “During the past month, how many times per week did you usually consume Tibetan butter tea or other salted beverages, such as salty soda?” Because Tibetan butter tea is the predominant traditional salted beverage in Xizang, it was used to assess its consumption frequency. Response options were never, less than once per week, once per week, 2–4 days per week, 5–6 days per week, once per day, and more than once per day. For analysis, responses were grouped as never, occasionally (less than once per week or once per week), frequently (2–4 days per week or 5–6 days per week), and daily (once per day or more than once per day). The never group served as the reference category in all regression models.

### 2.3. Covariates

The covariates included sex, school type, birth order, paternal education, maternal education, family type, family economic status, parental partnership, insufficient sleep, and insufficient physical activity.

Sex was recorded as male or female. School type was classified as either junior or senior. Birth order was categorized as first, second, or third or later. Paternal and maternal education were each grouped as primary school or below, middle/high school, or college and above. Family type was categorized as core or other. Family economic status was classified as good, average, or poor. Parental partnership was classified as excellent, good, or normal and below. Sleep duration was calculated as the weighted average of school-day and non-school-day sleep duration using a 5:2 weighting. Insufficient sleep was defined using age-specific thresholds [23]. Physical activity was assessed by asking students to report the number of days in the previous week on which they engaged in at least 60 min of moderate-to-vigorous physical activity. Insufficient physical activity was defined as fewer than 3 days per week [24].

### 2.4. Statistical Analysis

Participant characteristics were summarized as frequencies and percentages. Pearson’s chi-square tests were used to compare characteristics across school type, Tibetan butter tea consumption frequency, and cognitive performance group. Logistic regression models were used to estimate the association between the frequency of Tibetan butter tea consumption and low cognitive performance. Effect estimates were reported as odds ratios (ORs) with 95% confidence intervals (CIs). Two models were fitted. Model 1 estimated the unadjusted association between the frequency of Tibetan butter tea consumption and low cognitive performance. Model 2 adjusted for sex, school type, birth order, paternal education, maternal education, family type, family economic status, parental partnership, insufficient sleep, and insufficient physical activity. Cluster-robust standard errors were used to account for the correlation of responses among students within the same school or survey site. To examine whether the association differed by sex, analyses were repeated separately among female and male students. All statistical tests were two-sided, and *p* < 0.05 was considered statistically significant. All statistical analyses were performed using Stata/SE 18.0 (StataCorp LLC, College Station, TX, USA).

### 2.5. Ethical Approval and Consent to Participate

The study was approved by the Medical Ethics Committee of Shanghai Institute of Biomedical and Pharmaceutical Technologies. All procedures were conducted in accordance with the Declaration of Helsinki. Written informed consent was obtained from each participating student and from a parent or legal guardian. Participation was voluntary, and all data were de-identified before analysis.

## 3. Results

### 3.1. Study Participants

As shown in Table 1, the final analytic sample comprised 7967 Tibetan secondary school students (4433 females, 3534 males). Low cognitive performance did not differ by sex (male: 23.29%; female: 23.55%; *p* = 0.783). Butter tea consumption frequency, however, differed between male and female students (*p* < 0.001): daily consumption was more common among males (19.19% vs. 12.18%), whereas occasional consumption was more common among females (48.39% vs. 39.22%). Sex groups also differed across school type, maternal education, family type, family economic status, parental partnership, insufficient sleep, and insufficient physical activity (Table 1).

### 3.2. Differences in Participant Characteristics Across Butter Tea Consumption Frequency and Cognitive Performance Groups

Butter tea consumption frequency varied by sex, school type, paternal education, family economic status, insufficient sleep, and insufficient physical activity (all *p* < 0.05) (Table 2). Daily consumption was more common among males (19.19% vs. 12.18%) and junior students (21.50% vs. 10.38%). Students with insufficient sleep or insufficient physical activity were less likely to report daily consumption. No significant differences were observed by birth order, maternal education, family type, or parental partnership.

Cognitive performance groups also differed by butter tea consumption frequency (*p* < 0.001) (Table 3). Low cognitive performance was most prevalent among daily (29.56%) and never consumers (26.34%) and less prevalent among occasional (22.18%) and frequent consumers (20.39%). Low cognitive performance further varied by birth order, parental education, family type, family economic status, parental partnership, and insufficient sleep (all *p* < 0.05), but not by sex, school type, or insufficient physical activity.

### 3.3. Association Between Tibetan Butter Tea Consumption Frequency and Low Cognitive Performance

In the overall sample, occasional and frequent consumption were associated with lower odds of low cognitive performance in both crude (OR = 0.80; 95% CI, 0.68–0.93 and OR = 0.72; 95% CI, 0.60–0.85, respectively) and adjusted models (aOR = 0.79; 95% CI, 0.70–0.88 and aOR = 0.71; 95% CI, 0.55–0.90). Daily consumption was not significantly associated with low cognitive performance (Table 4).

In sex-stratified analyses, the pattern was generally consistent. Frequent consumption was associated with lower adjusted odds of low cognitive performance in both females (aOR = 0.74; 95% CI, 0.58–0.93) and males (aOR = 0.68; 95% CI, 0.49–0.93). Occasional consumption was statistically significant among females (aOR = 0.76; 95% CI, 0.64–0.90) but not among males (aOR = 0.83; 95% CI, 0.63–1.08). Daily consumption was not significantly associated with low cognitive performance in either sex (Table 4).

## 4. Discussion

To our knowledge, this is the first study to examine the association between Tibetan butter tea and cognitive performance among high-altitude Tibetan adolescents. In this school-based cross-sectional study of 7967 Tibetan secondary school students in Lhasa, occasional and frequent butter tea consumption were associated with lower odds of low cognitive performance compared with never consuming butter tea, whereas daily consumption showed no significant association. Sex-stratified analyses yielded a broadly similar pattern: frequent consumption was associated with lower odds in both sexes, while occasional consumption was significant only among females.

Although butter tea’s sodium and fat content would, from a nutrient-centered perspective, predict adverse cognitive outcomes [25,26,27], this reasoning overlooks that butter tea is not simply a vehicle for salt and fat; it is also a source of tea-derived bioactive compounds, hydration, and energy in a cold, high-altitude environment [7,8]. Our finding of lower odds of low cognitive performance among occasional and frequent consumers, together with the null result for daily consumption, suggests that the association was observed at occasional/frequent consumption but was not present at daily consumption and may therefore vary across consumption frequency. One possible explanation is that butter tea contains tea-derived bioactive compounds, including caffeine and L-theanine, which have been associated with improvements in selected short-term cognitive outcomes such as attention and vigilance [12,28]; thus, the cognitive benefit observed at moderate intake may be attributable to these tea-derived bioactives, whereas the null result at daily consumption may reflect a progressively greater sodium and fat load offsetting or attenuating this benefit. This pattern underscores the importance of evaluating traditional foods as integrated dietary exposures rather than inferring health effects from single components. Furthermore, prospective evidence indicates that tea intake and dementia risk exhibit a non-linear dose–response relationship, with protection strengthening up to a moderate level and not increasing further—or even diminishing—at higher intake [29].

No directly comparable study of Tibetan butter tea and cognition is available. Although tea-related studies provide biological plausibility for tea-specific cognitive effects [12,28], they cannot be directly extrapolated to the present study because Tibetan butter tea is a salted, butter-containing beverage and the study population consists of high-altitude adolescents, a group in whom no prior dietary cognition evidence exists. From a behavioral perspective, daily butter tea consumption may reflect broader dietary and lifestyle patterns that differ from those of occasional or frequent consumers. Because overall dietary quality, total energy intake, and other dietary behaviors were not measured, residual confounding by these unmeasured dietary factors cannot be excluded. The present findings, therefore, extend the tea–cognition literature into a novel dietary and population context, rather than merely replicating prior observations.

The sex-stratified findings were broadly consistent with the overall pattern: frequent consumption was associated with lower odds of low cognitive performance in both sexes. The sex difference lay in occasional consumption, which was statistically significant only among females. Two observations should be considered when interpreting this difference. First, male and female students differed markedly in consumption frequency: daily intake was more common among males, and occasional intake was more common among females, which may have affected the statistical precision and comparability of stratum-specific estimates. Second, adolescence is a period of sex-differentiated neurodevelopment, and reviews emphasize that these differences are complex and age-dependent rather than evidence of fixed cognitive disparities [30,31]. However, the available data do not include pubertal status, butter tea quantity, or preparation characteristics, making it difficult to distinguish biological, behavioral, and measurement-related explanations. These stratified results should therefore be regarded as exploratory and interpreted primarily to support the internal consistency of the main finding, rather than as evidence of a true sex difference.

This study has several limitations. First, the cross-sectional design precludes causal inference. As exposure and outcome were measured at a single time point, the temporal sequence is unknown, and reverse causation or residual confounding cannot be excluded; the observed associations should therefore be interpreted cautiously and not as evidence of a causal effect. Second, the exposure item referred to Tibetan butter tea together with other salted beverages. Although Tibetan butter tea is the dominant salted traditional beverage in Xizang, this wording may have partially captured other salted drinks, yielding exposure misclassification; although the misclassification is likely to have been largely non-differential with respect to cognitive performance, the extent and direction of the resulting bias cannot be determined. Third, the exposure was based on self-reported frequency, which may be prone to recall bias in adolescents, and it was not validated against objective dietary assessment methods; because actual intake was not quantified, a true dose–response relationship could not be evaluated. Future studies using validated, quantitative instruments specifically designed to assess Tibetan butter tea intake are needed to further validate these findings. Fourth, RSPM primarily assesses nonverbal reasoning and fluid intelligence [20,32], and our findings may not generalize to other cognitive domains. Fifth, information on overall dietary habits (e.g., breakfast consumption, dairy intake, dietary quality, and total energy intake) was not assessed, so residual confounding by broader dietary patterns cannot be ruled out; the observed associations may therefore partly reflect dietary patterns associated with butter tea consumption rather than butter tea itself. Finally, this study did not control for seasonal variation in butter tea consumption or cognitive performance, and thus their potential influence on the estimated associations could not be fully excluded.

## 5. Conclusions

In this cross-sectional study of Tibetan adolescents in Xizang, moderate butter tea consumption (occasional to frequent) was linked to lower odds of low cognitive performance, whereas daily consumption did not show a similar association. These findings suggest that the cognitive effects of Tibetan butter tea should be assessed as a culturally embedded whole-dietary exposure, rather than being inferred from its isolated nutritional components, such as salt or fat content. This finding suggests that school-based nutrition education and public health guidance in high-altitude Tibetan regions should take into account both cultural context and dietary moderation. It should be emphasized that, as this study employed a cross-sectional design with exposure measured through self-reported frequency, causal inference cannot be established, and detailed information on intake amounts, composition, and overall dietary patterns was lacking. Therefore, these findings require further verification through longitudinal studies and more refined dietary assessments.

## Figures and Tables

**Table 1:** Demographic characteristics by sex, *n* (%).

	Male	Female	*p* Value
	(N = 3534)	(N = 4433)	
Cognitive group			
Q2–Q4	2711 (76.71)	3389 (76.45)	0.783
Q1	823 (23.29)	1044 (23.55)	
Frequency of Tibetan butter tea consumption			
Never	467 (13.21)	672 (15.16)	<0.001
Occasionally	1386 (39.22)	2145 (48.39)	
Frequently	1003 (28.38)	1076 (24.27)	
Daily	678 (19.19)	540 (12.18)	
School type			
Junior school	1622 (45.90)	1895 (42.75)	0.005
Senior school	1912 (54.10)	2538 (57.25)	
Birth order			
First	1918 (54.37)	2289 (51.66)	0.053
Second	932 (26.42)	1228 (27.71)	
Third or later	678 (19.22)	914 (20.63)	
Paternal education level			
Primary school and below	1878 (53.20)	2410 (54.39)	0.187
Middle/High school	1078 (30.54)	1366 (30.83)	
College and above	574 (16.26)	655 (14.78)	
Maternal education level			
Primary school and below	2301 (65.15)	2961 (66.81)	0.001
Middle/High school	722 (20.44)	953 (21.50)	
College and above	509 (14.41)	518 (11.69)	
Family type			
Core family	2170 (61.40)	2615 (59.00)	0.030
Others	1364 (38.60)	1817 (41.00)	
Family economy			
Good	1283 (36.34)	1588 (35.85)	<0.001
Average	1830 (51.83)	2446 (55.21)	
Poor	418 (11.84)	396 (8.94)	
Parent partnership			
Excellent	2642 (74.82)	3017 (68.09)	<0.001
Good	585 (16.57)	895 (20.20)	
Normal and below	304 (8.61)	519 (11.71)	
Insufficient sleep			
No	1966 (56.00)	2048 (46.43)	<0.001
Yes	1545 (44.00)	2363 (53.57)	
Insufficient physical activity			
No	2126 (60.18)	2206 (49.76)	<0.001
Yes	1407 (39.82)	2227 (50.24)	

**Table 2:** Distribution of Tibetan butter tea categories and associated factors, *n* (%).

Variable	Frequency of Tibetan Butter Tea Consumption				Chi-Square	*p* Value
	Never	Occasionally	Frequently	Daily		
Sex					118.31	<0.001
Male	467 (13.21)	1386 (39.22)	1003 (28.38)	678 (19.19)		
Female	672 (15.16)	2145 (48.39)	1076 (24.27)	540 (12.18)		
School type					190.11	<0.001
Junior school	453 (12.88)	1427 (40.57)	881 (25.05)	756 (21.50)		
Senior school	686 (15.42)	2104 (47.28)	1198 (26.92)	462 (10.38)		
Birth order					2.71	0.845
First	607 (14.43)	1873 (44.52)	1099 (26.12)	628 (14.93)		
Second	313 (14.49)	934 (43.24)	569 (26.34)	344 (15.93)		
Third or later	216 (13.57)	721 (45.29)	410 (25.75)	245 (15.39)		
Paternal education level					12.65	0.049
Primary school and below	600 (13.99)	1915 (44.66)	1098 (25.61)	675 (15.74)		
Middle/High school	340 (13.91)	1069 (43.74)	647 (26.47)	388 (15.88)		
College and above	198 (16.11)	546 (44.43)	333 (27.10)	152 (12.37)		
Maternal education level					10.79	0.095
Primary school and below	728 (13.84)	2353 (44.72)	1354 (25.73)	827 (15.72)		
Middle/High school	254 (15.16)	727 (43.40)	433 (25.85)	261 (15.58)		
College and above	156 (15.19)	451 (43.91)	291 (28.33)	129 (12.56)		
Family type					0.44	0.932
Core family	682 (14.25)	2111 (44.12)	1251 (26.14)	741 (15.49)		
Others	456 (14.34)	1420 (44.64)	828 (26.03)	477 (15.00)		
Family economy					17.80	0.007
Good	422 (14.70)	1304 (45.42)	714 (24.87)	431 (15.01)		
Average	567 (13.26)	1892 (44.25)	1154 (26.99)	663 (15.51)		
Poor	147 (18.06)	333 (40.91)	210 (25.80)	124 (15.23)		
Parent partnership					5.51	0.481
Excellent	807 (14.26)	2515 (44.44)	1460 (25.80)	877 (15.50)		
Good	201 (13.58)	649 (43.85)	414 (27.97)	216 (14.59)		
Normal and below	131 (15.92)	363 (44.11)	204 (24.79)	125 (15.19)		
Insufficient sleep					26.69	<0.001
No	539 (13.43)	1747 (43.52)	1036 (25.81)	692 (17.24)		
Yes	597 (15.28)	1765 (45.16)	1028 (26.31)	518 (13.25)		
Insufficient physical activity					48.66	<0.001
No	565 (13.04)	1841 (42.50)	1172 (27.05)	754 (17.41)		
Yes	574 (15.80)	1689 (46.48)	907 (24.96)	464 (12.77)		

**Table 3:** Distribution of cognitive groups and associated factors, *n* (%).

Variable	Cognitive Group		Chi-Square	*p* Value
	Q2–Q4	Q1		
Frequency of Tibetan butter tea consumption			44.63	<0.001
Never	839 (73.66)	300 (26.34)		
Occasionally	2748 (77.82)	783 (22.18)		
Frequently	1655 (79.61)	424 (20.39)		
Daily	858 (70.44)	360 (29.56)		
Sex			0.08	0.783
Male	2711 (76.71)	823 (23.29)		
Female	3389 (76.45)	1044 (23.55)		
School type			0.27	0.601
Junior school	2683 (76.29)	834 (23.71)		
Senior school	3417 (76.79)	1033 (23.21)		
Birth order			14.55	<0.001
First	3288 (78.16)	919 (21.84)		
Second	1635 (75.69)	525 (24.31)		
Third or later	1172 (73.62)	420 (26.38)		
Paternal education level			45.40	<0.001
Primary school and below	3241 (75.58)	1047 (24.42)		
Middle/High school	1822 (74.55)	622 (25.45)		
College and above	1032 (83.97)	197 (16.03)		
Maternal education level			44.85	<0.001
Primary school and below	3972 (75.48)	1290 (24.52)		
Middle/High school	1255 (74.93)	420 (25.07)		
College and above	871 (84.81)	156 (15.19)		
Family type			6.87	0.009
Core family	3615 (75.55)	1170 (24.45)		
Others	2484 (78.09)	697 (21.91)		
Family economy			7.89	0.019
Good	2149 (74.85)	722 (25.15)		
Average	3323 (77.71)	953 (22.29)		
Poor	626 (76.90)	188 (23.10)		
Parent partnership			27.30	<0.001
Excellent	4291 (75.83)	1368 (24.17)		
Good	1205 (81.42)	275 (18.58)		
Normal and below	600 (72.90)	223 (27.10)		
Insufficient sleep			53.29	<0.001
No	2936 (73.14)	1078 (26.86)		
Yes	3130 (80.09)	778 (19.91)		
Insufficient physical activity			1.58	0.208
No	3293 (76.02)	1039 (23.98)		
Yes	2806 (77.22)	828 (22.78)		

**Table 4:** Association of Tibetan butter tea consumption with low cognition (overall and by sex).

Variable	Model 1 *		Model 2 *	
	Crude OR (95% CI)	*p* Value	Adjusted OR (95% CI)	*p* Value
Total				
Never	Reference	NA	Reference	NA
Occasionally	0.80 (0.68–0.93)	0.004	0.79 (0.70–0.88)	<0.001
Frequently	0.72 (0.60–0.85)	<0.001	0.71 (0.55–0.90)	0.004
Daily	1.17 (0.98–1.41)	0.082	1.09 (0.69–1.74)	0.708
Male				
Never	Reference	NA	Reference	NA
Occasionally	0.84 (0.66–1.07)	0.150	0.83 (0.63–1.08)	0.160
Frequently	0.70 (0.54–0.90)	0.007	0.68 (0.49–0.93)	0.018
Daily	1.18 (0.91–1.54)	0.212	1.11 (0.58–2.12)	0.758
Female				
Never	Reference	NA	Reference	NA
Occasionally	0.77 (0.63–0.94)	0.010	0.76 (0.64–0.90)	0.001
Frequently	0.74 (0.59–0.93)	0.008	0.74 (0.58–0.93)	0.011
Daily	1.18 (0.92–1.52)	0.192	1.09 (0.75–1.57)	0.655

Note: * Model 1: Crude. Model 2: Overall model adjusted for sex, school type, birth order, parental education, family type, family economy, parental relationship, sleep, and physical activity; sex-stratified models adjusted for the same covariates except sex. Abbreviation: NA = not applicable; OR = odds ratio; CI = confidence interval.

## Data Availability

The data presented in this study are available on request from the corresponding author due to privacy and ethical restrictions.
